# A New *In Silico* Comparison of the Relative Affinity of Enantiomeric Chloroquine (CQ) and Hydroxychloroquine (HCQ) for ACE2

**DOI:** 10.3390/ph18070982

**Published:** 2025-06-30

**Authors:** Carlos Naranjo-Castañeda, Marco A. García-Revilla, Eusebio Juaristi

**Affiliations:** 1 Department of Chemistry, Centro de Investigación y de Estudios Avanzados del Instituto Politécnico Nacional, Avenida Instituto Politécnico Nacional No. 2508, San Pedro Zacatenco, Ciudad de México 07360, Mexico; carlos.naranjo@cinvestav.mx; 2 Department of Chemistry, Universidad de Guanajuato, Noria Alta S/N, Guanajuato 36050, Mexico; 3 El Colegio Nacional, Donceles # 104, Centro Histórico, Ciudad de México 06020, Mexico

**Keywords:** COVID-19, ACE2, chloroquine, hydroxychloroquine, enantiodifferentiation, molecular docking, molecular dynamics, MM-PBSA

## Abstract

**Background/Objectives**: Chloroquine (CQ) and hydroxychloroquine (HCQ) have been the subject of debate in the treatment of COVID-19 due to the lack of conclusive evidence regarding their efficacy and safety. Our study aims to investigate the molecular interaction between the enantiomers of CQ and HCQ with angiotensin-converting enzyme 2 (ACE2), focusing on the binding mechanism, affinity, and selectivity. **Methods**: We used in silico methods, including molecular docking, molecular dynamics, and binding free energy calculations using the MM-PBSA method, to evaluate the interaction between the enantiomers of CQ and HCQ with ACE2. **Results**: We identified three main interaction sites on ACE2 (α, β, and γ) with distinct characteristics based on the pocket size, hydrophilic/hydrophobic characteristics, and affinity energy. We observed that protonation states and ionic strength significantly influence the binding affinity and specificity. In particular, the selectivity of the β-site, characterized by its smaller size and hydrophilic residues, is preferential for species with the (*R*) configuration, whereas the α and γ binding sites, with a larger size and amphiphilic residues, have greater affinity for the (*S*) enantiomer of CQ and HCQ. Furthermore, ionic strength can affect ligand binding by modulating electrostatic interactions, molecular conformation, solvation, and the stability of the complex. **Conclusions**: Our findings reveal that protonation states and the ionic strength substantially impact the binding affinity and specificity, regulated by spatial and polar–electrostatic complementarity, as well as hydrophobic contributions. These results suggest that understanding the interaction between CQ and HCQ enantiomers with ACE2 could be useful for the design of novel therapies against COVID-19.

## 1. Introduction

The interaction between the enantiomers of chloroquine (CQ) and hydroxychloroquine (HCQ) with the angiotensin-converting enzyme 2 (ACE2) is crucial in the search for effective therapies against COVID-19, given that ACE2 acts as a specific receptor for severe acute respiratory syndrome coronavirus 2 (SARS-CoV-2), facilitating its entry into host cells. Although health authorities do not recommend the administration of these drugs due to the lack of conclusive evidence on their efficacy and safety, the debate about the effectiveness of the enantiomeric forms of CQ and HCQ on ACE2 remains intense [1]. Despite the lack of consensus on their efficacy in hospitalized patients, CQ and HCQ remain interesting for chemoprevention [2,3].

The recommendation of regulatory agencies to use enantiopure chiral drugs instead of racemates has motivated the study of these drugs as individual enantiomers [4,5]. However, previous studies have reported conflicting results regarding the binding efficiency of the (*R*)- and (*S*)-enantiomers of HCQ to ACE2: The (*R*)-enantiomer of HCQ has been shown to exhibit greater efficacy compared to the (*S*)-enantiomer, with significantly higher antiviral activity (EC50 = 3.05 μM vs. 5.38 μM) [6,7]. However, it has also been reported that the (*S*)-enantiomer of HCQ may exhibit higher acute toxicity in vivo [8]. The pharmacokinetics of HCQ are also enantioselective, with higher blood concentrations of the (*R*)-enantiomer and stereoselective metabolism resulting in higher concentrations of (*S*)-enantiomer metabolites [9,10]. In contrast, the ACE2 enzyme has also been reported to prefer the (*S*)-enantiomer of HCQ [1,11]. Although the specific interaction site on ACE2 remains a matter of debate due to the existence of multiple sites and the complex protein reorganization dependent on allosteric movements, progress has been made in understanding the interaction between CQ and HCQ with ACE2. However, the binding mechanism and the molecular structure underlying this binding remain poorly understood. In addition, there is no direct evidence of the binding between the ACE2 enzyme and HCQ or CQ.

In particular, it is unknown which binding site on ACE2 is most relevant for chiral species and whether a cooperative effect or an allosteric interaction between the different sites determines the preference for one ligand over the other. Therefore, this work investigates the enantiodiscrimination in the binding of CQ and HCQ to ACE2 and its potential contribution to therapeutic efficacy, with the aim of providing a deeper understanding of the molecular interaction underlying ligand binding and the stability of the formed complex.

**Figure 1:**
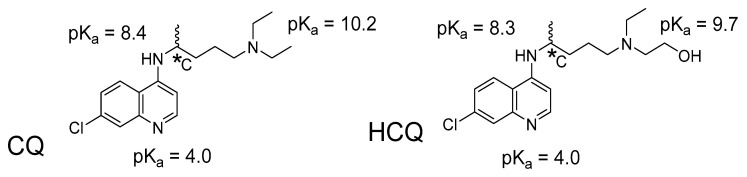
Basic functional groups in CQ and HCQ, and pK_a_ values for the corresponding conjugated acids. *C indicates the center of chirality.

Indeed, a salient characteristic of CQ and HCQ inhibitors addressed in the present computational study is that these ligands incorporate a chiral carbon atom that gives rise to the enantiomers (*R*)-CQ, (*S*)-CQ, (*R*)-HCQ, and (*S*)-HCQ, which are distinct molecules with potentially different pharmacological activity. All four species behave as weak amphiphilic bases [12]; in particular, HCQ incorporates three basic functional groups with associated pK_a_ values of 4.0 (aromatic nitrogen), 8.3 (secondary aniline-type nitrogen), and 9.7 (tertiary amine nitrogen) for their conjugated acids. CQ exhibits essentially similar pK_a_ values of 4.0 (aromatic nitrogen), 8.4 (secondary aniline-type nitrogen), and 10.2 (tertiary nitrogen) (Figure 1) [13,14]. The corresponding acid/base properties are of interest for exploring the resulting activity of these drugs against ACE2.

Previously [15], CQ and HCQ had been tested against SARS-CoV coronavirus, and indeed, they were found to be effective in inhibiting viral invasion; thus, it was decided to explore these drugs against the SARS-CoV-2 coronavirus since their structures present a similarity of 97.4%. SARS-CoV and SARS-CoV-2 present a similar spherical shape as a result of four constituent proteins that induce spikes (S), envelope (E), membrane (M), and nucleocapsid (N) conformations. Among these structural features, the spikes stand out since they are directly responsible for viral invasion [15,16] and correspond to glycoproteins found on the surface of the virus, consisting of a receptor-binding domain (RBD) that exhibits a high affinity for angiotensin-converting enzyme 2 (ACE2). In this sense, ACE2 is found on the surface of human cells, which incorporates a glycoprotein that binds to the cytoplasmic membrane type I. ACE2 consists of 805 amino acids distributed in a catalytic domain (with subdomains I and II) and a protease domain (PD) [17]. For viral invasion to occur, an assembly must be formed between viral RBD and the PD region in ACE2 [16].

**Figure 2:**
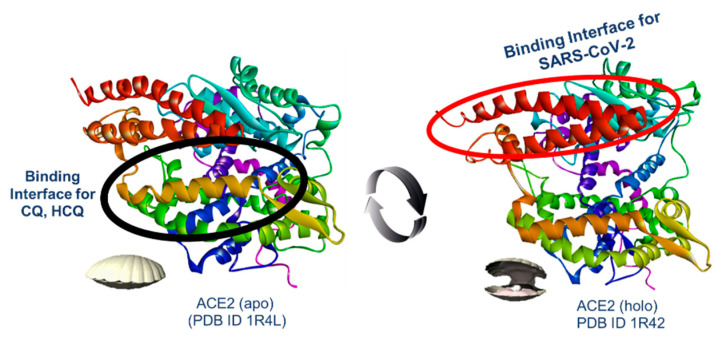
ACE2 conformational changes are induced during the binding process (opening and closing movement). **Left**, closed conformation (black circle: binding interface for CQ and HCQ); **right**, open conformation (red circle: binding interface for SARS-CoC-2).

In silico studies shed light on how CQ and HCQ interact with allosteric sites distal to the active site in ACE2 rather than coupling to the PD surface [16,18]. However, the area of interaction is quite extensive and it has not been possible to establish with certainty which amino acid residues on ACE2 exhibit greater affinity. In this regard, it is known that interactions taking place during binding cause conformational changes in the ACE2 receptor, which have been described as being similar to the movements made by a clam (an opening and closing movement) (Figure 2) [19]. In this sense, several reports indicate that spikes bind less efficiently to ACE2 receptors when coupled with a natural inhibitor relative to the native form [20]. For example, binding involving spikes is affected by the incorporation of inhibitor-selective MLN-4760 [21] within the active site since the ACE2 receptor adopts a closed conformation (holo-ACE2, http://www.rcsb.org, accessed on 25 June 2025, ID 1R4L), decreasing its efficiency by up to 1.48 times concerning binding, which takes place with the free enzyme that adopts an open conformation (apo-ACE2, PDB ID 1R42) [22].

Therefore, locating the main region of the interaction of CQ and HCQ in ACE2 is of paramount importance. In addition, determining the relative impact of the stereochemical configuration of enantiomeric protonated CQ and HCQ on the conformational stability of the receptor protein is essential since the binding of these drugs can induce the enzyme to adopt a closed conformation that, in principle, would prevent binding with viral spikes during infection (Figure 2).

**Figure 3:**
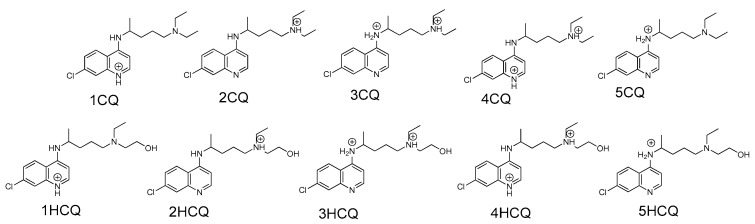
Likely protonated states in the (*R*) and (*S*) enantiomers of CQ and HCQ at physiological pH. Ten different protonated structures were considered for CQ (1CQ-5CQ) and ten for HCQ (1HCQ-5HCQ) (both enantiomeric forms), in addition to four neutral species (Figure 1). Note: the representation in the figure includes both enantiomeric forms, although the stereochemistry is not shown explicitly, a circle with a plus sign is used to display the charge over the nitrogen atoms.

The present study was carried out systematically, in which the salient regions of the interaction were initially determined by considering the more feasible protonation states of both enantiomers, which were numbered as depicted in Figure 3. Subsequently, the interaction and molecular affinity of (*R*)-CQ, (*S*)-CQ, (*R*)-HCQ, and (*S*)-HCQ in specific regions within ACE2 were examined to establish the enzymatic sites with greater affinity. Finally, molecular dynamics (MD) simulations were used to evaluate the reactivity and stability of the resulting complexes and the associated free energy differences according to the Poisson–Boltzmann Surface Area method of Molecular Mechanics (MM-PBSA).

## 2. Results and Discussion

### 2.1. Pharmacological Potential of Ligands and Prediction of the Main Interaction Regions

**Figure 4:**
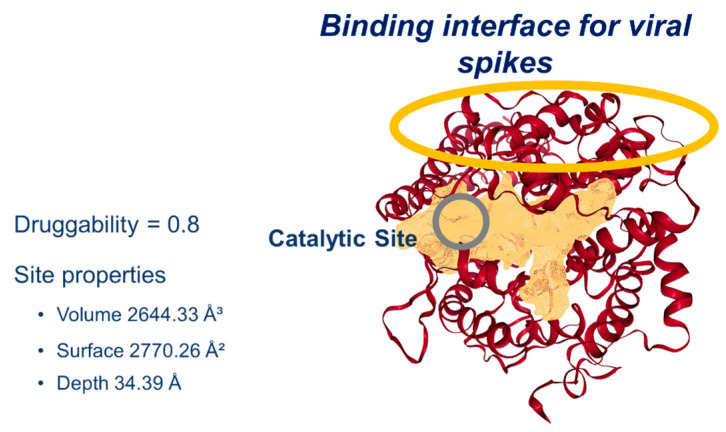
Calculated ACE2 interaction zone for CQ and HCQ (yellow mesh), the yellow and gray circles delineate the binding interface and the catalytic site, respectively.

In silico studies identified the main areas of interaction between CQ and HCQ drugs and the ACE2 receptor, which occur at the periphery of the enzyme’s catalytic site [16,18]. Nevertheless, the above reports do not specify the relative affinity between those different binding regions. As a first approximation, in the present work, the DoGSiteScorer (a web server for automatic binding site prediction) [23] was used to detect and analyze potential binding pockets in ACE2 toward (*R*)-CQ, (*S*)-CQ, (*R*)-HCQ, and (*S*)-HCQ substrates, as well as to estimate the “druggability,” that is the capacity of the acceptor protein to bind potential ligands or drugs [24]. In this way, the most likely interaction region was identified with a pharmacological potential of 0.8, corresponding to a remarkable value compared to other pockets within ACE2. The preferred binding site comprises a volume of 2644.33 Å^3^, with an area of 2770.26 Å^2^ and a depth of 34.39 Å, reaching beyond the region of the catalytic site, which agrees with the binding sites suggested in the literature. In addition, the prominent amino acids identified at the binding site were classified as polar (42% of them), non-polar (38% of them), and charged (20% of them) amino acids ubicated in the region of subdomain I that is associated with the hinge-bending motion of ACE2, which is fundamental in ACE2 affinity for viral spikes and subsequent catalysis (Figure 4) [19].

### 2.2. Molecular Docking

The molecular docking study of the inhibitors of interest was carried out systematically. First, the possibility of finding binding sites throughout the protein surface was explored by combining three descriptors: the pocket volume, receptor hydrophobicity, and binding energy. Next, localized molecular docking was performed, where the specific affinity of the ligands for the binding sites was estimated. In addition, flexible docking was performed to implicitly allow a certain degree of interpenetration between the receptor protein and the docked ligand. Furthermore, the consequences of solvent participation during ligand binding (by flexible/hydrated docking) were evaluated by considering the presence of charged sites within the ligand. Furthermore, the effect of the enantiomers on the racemic mixture was evaluated by simultaneous coupling. Finally, we present the validation of the method by comparing the relative efficiency of HCQ and MLN-4760.

**Figure 5:**
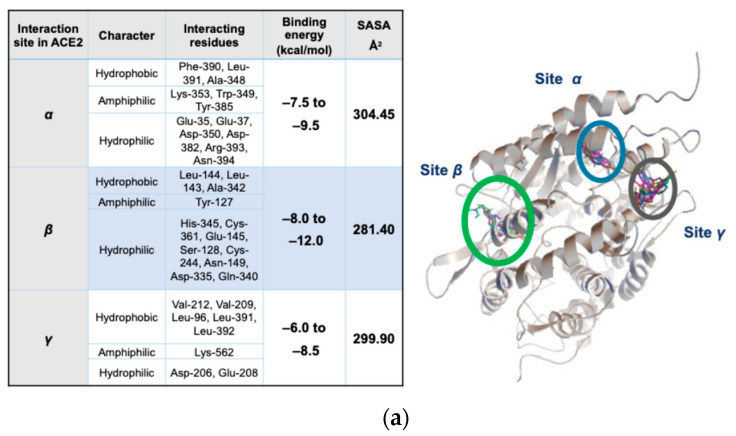

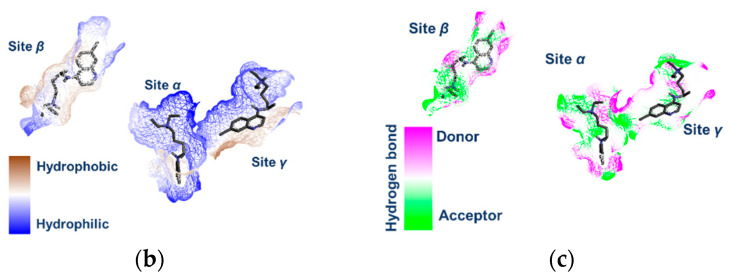
(**a**) Principal interaction sites (α, β, and γ). Prominent amino acid residues involved in the binding interactions are highlighted, as well as the binding free energy score range and solvent-accessible surface areas (SASA) (Green circle: inset on β-site. Yellow circle: inset on α-site. Black circle: inset on γ-site.). (**b**) Surface characteristics and the corresponding hydrophilic/hydrophobic character. (**c**) Surface characteristics highlight groups that participate in hydrogen bonding.

Initially, a blind coupling evaluation was performed to identify the regions in ACE2, with a greater affinity towards (*R*)-CQ, (*S*)-CQ, (*R*)-HCQ, and (*S*)-HCQ. At this stage, plausible protonation states were considered and contrasted. Each ligand docking to ACE2 afforded multiple conformations of the coupled ligand and the corresponding binding scores. Root mean square deviation (RMSD) for the coupled conformations was calculated, and a relatively large RMSD limit value of 8.0 Å was selected upon the consideration of the large binding site determined above and the significant number of rotating bonds in the ligands. It was generally observed that the chosen protonation states of the ligands do favor the three distinct binding sites previously identified in the literature, and labeled as α [18], β [15], and γ [16] (Figure 5a).

A localized docking strategy with the protonated states of the stereoisomers confirmed these binding sites. The most prominent binding site was identified as β, with binding energy values ranging from −8 to −12 kcal/mol. Once the three binding sites had been characterized, the hydrophilic/hydrophobic character of the most relevant peptide residues in those sites was identified. In particular, for the β-site, the presence of hydrophobic zones that can interact with the aromatic segment of the ligands can be appreciated (Figure 5b). In addition, at the β binding site, the presence of polar compact zones was established (SASA: 281.40 Å^2^), whereas in the α and γ sites, the binding pockets are more spacious, which allows the greater mobility of the bound ligands, which exhibit accordingly weaker adhesion (Figure 5c). In the CQ/ACE2 and HCQ/ACE2 systems, the smaller β site size compared to α and γ sites forced the ligand to adopt a reduced number of effective conformations that met the structural requirements for an effective interaction with the receptor. Thus, for flexible ligands, the affinity between species is regulated by the degree of complementarity and the energy cost associated with the conformational reorganization [25,26] that allows the bioactive conformation of the coupled ligand. This fact can be extrapolated to the idea that the average free energy is lower for α and γ sites than for β-site.

From the data obtained by localized docking, it is concluded that the selectivity of the β-site is preferential for the *R-configured* species (see Table 1 and Table 2), giving rise to a higher level of binding free energy relative to that exhibited by the (*S*) isomer. Likewise, the inhibition constant (k_i_) of the ligand–protein complex in the β-site was lower, k_i_ ≈ 0.150 μM for the (*R*) enantiomers of CQ and HCQ, which means that the ligand/ACE2 complex was efficient enough to occupy 50% of the binding site even when present in a lower concentration. Statistically, the percentage of effective poses was 8–10% of 150 conformers associated with an RMSD of less than 2 Å, indicating that many conformers can fit within the binding β-site. In contrast, the larger α and γ binding sites exhibit a higher affinity for the (*S*) enantiomer of CQ and HCQ as they exhibit a lower inhibition constant and % pose greater than 19%. These facts make this study enjoyable and lead us to specify the ligand–enzyme complex’s activity further.

Considering the physiological pH (≈7.2) [27], CQ and HCQ are likely to exist in a specific protonation state, with the monoprotonated state at the tertiary amino nitrogen being the most plausible due to its pKa being compatible with the physiological pH [28]. This monoprotonated species is probably the predominant form in the extracellular physiological environment and plays an important role in the interaction with its target, so it was selected as the most relevant state for the study of CQ and HCQ binding. In particular, attention was paid to the (*R*) and (*S*) stereoisomers of the monoprotonated species 2CQ and 2HCQ, since they are the most likely to be present in the extracellular environment at a physiological pH.

**Figure 6:**
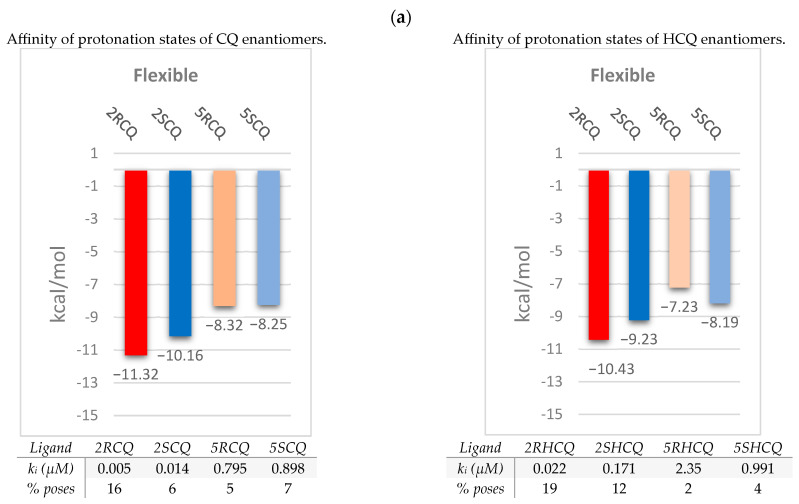

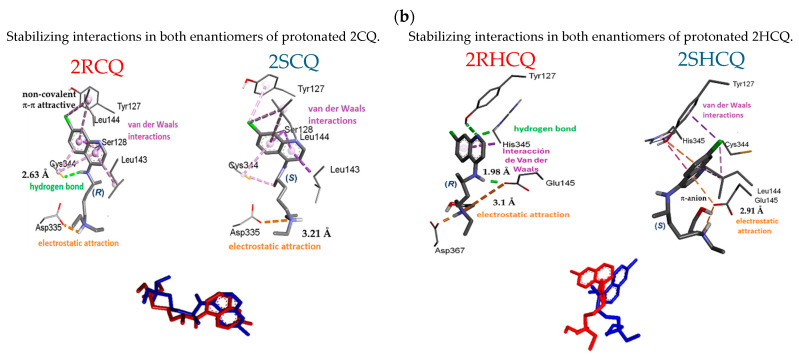
Results from the flexible coupling of ligands CQ and HCQ at ACE2’s β-site. (**a**) Binding affinity of the protonated species at the tertiary amino group (2RCQ, 2SCQ, 2RHCQ, and 2SHCQ) and the species in the protonated aniline-type secondary nitrogen (5RCQ, 5SCQ, 5RHCQ, and 5SHCQ). (**b**) Most prominent non-bonded interactions in the lowest energy conformations for protonated 2RCQ, 2SCQ, 2RHCQ, and 2SHCQ, and superposition of the enantiomers of 2CQ and 2HCQ (red enantiomer *R* and blue enantiomer *S*). Calculated inhibition constants, k_i_, in micro molar units, µM, are placed in the shadow data.

Flexible coupling [29] was then performed to find the best binding conformational poses of the protonated ligands 2CQ and 2HCQ at the β binding site. Special attention was paid to interactions involving the residues of Ser-128, Glu-145, Cys-344, Asp-335, and Asp-367, given their ability to participate in hydrogen bonding as well as in electrostatic interactions via so-called “salt bridges” with protonated ligands [30,31]. The optimized conformation of the bound ligands suggested a distinction between the two conformational populations: (1) *Bent conformation* due to dominant intermolecular interactions between the tertiary amine nitrogen and the aniline-type amino group with one particular amino acid residue in ACE2. (2) *Extended conformation* exhibits intermolecular interactions between the tertiary amino group and the secondary aniline-type amino group and two distinct amino acid residues at the β-site that are distant (Figure 6b).

Additionally, Figure 6b includes the data collected from the flexible coupling study of the protonated (*R*)- and (*S*)-stereoisomers of 2CQ, 5CQ, 2HCQ, and 5HCQ at the β-site. For CQ as the substrate, the (*R*)-*configured* species, 2RCQ, is found to adopt an *extended conformation* so that the quinoline ring fits well within the hydrophobic cavity of the pocket and is stabilized by a non-covalent π–π attractive interaction with the Tyr-127 amino acid residue at the β-site. In addition, this *extended conformation* is stabilized by electrostatic attractive interactions with the Asp-335 residue (Figure 6b). A salient observation is the formation of a hydrogen bond between the N-secondary amino group and the thiol group in Cys-344 (NH–S distance = 2.63 Å). These stabilizing interactions with the (*R*) enantiomer provide a binding free-energy score value of −11.32 kcal/mol, k_i_ = 0.005 µM, and 16% number of poses. In contrast, the (*S*)-*configured* enantiomer 2SCQ exhibited an affinity of −10.13 kcal/mol, k_i_ = 0.014 µM, and 6% number of poses, also in an *extended conformation* induced by an electrostatic attraction between the Asp-335 carboxylate group and the protonated N-tertiary amino group (O^−^–HN^+^ distance = 3.21 Å). These data suggest the greater stability of the (*R*)-*configured* 2RCQ complex because it presents a more negative binding free-energy value and a better inhibition constant. Interestingly, for CQ ligands protonated in the aniline-type nitrogen (5RCQ and 5SCQ), which are less likely to be populated at a physiological pH, no significant difference was observed in their inhibition constant and free-energy score values: −8.32 and −8.28 kcal/mol, respectively.

In the case of HCQ ligands, the (*R*) enantiomer 2RHCQ adopts a *bent conformation* as a consequence of two convergent stabilizing interactions: one hydrogen bond between the carboxylate group of Glu-145 and the secondary nitrogen in HCQ (O^−^–H-N distance = 1.98 Å) and one electrostatic interaction between the same residue Glu-145 and the protonated tertiary nitrogen in the HCQ ligand (O^−^–HN^+^ distance = 3.1 Å). These stabilizing interactions provide a free bond energy value of −10.43 kcal/mol, k_i_ = 0.022 µM, and 19% number of poses.

Although the (*S*) enantiomer 2SHCQ also adopts a *bent conformation* converging on the amino acid residue Glu-145, the responsible attractive interactions seem to be weaker, with one of the type π–anion involving the quinoline ring and the carboxylate group on the amino acid residue (π–O^−^ distance = 2.91 Å), reflected in a binding energy score value of −9.23 kcal/mol, k_i_ = 0.171 μM, and 12% number of poses.

Furthermore, Figure 6b shows the superposition of the enantiomers, revealing the distribution of the ligand atoms within the β-site, and highlights the spatial differences in the calculated conformations for each enantiomer, providing insight into the spatial constraint of the ligands within the binding site (SASA = 281.40 Å^2^).

The molecular docking study features a rigorous statistical analysis to support the results, including F and Mann–Whitney tests, as well as confidence intervals to estimate the accuracy of the binding free energies. The F test (with an alpha parameter equal to 0.05) suggests that the variation in the data distribution between the enantiomer pairs is equal, although caution should be exercised due to unequal sample sizes and the potential non-normality of the data (see Tables S2–S7 in the Supporting Information). The 95% confidence intervals for each enantiomer show no overlap between the binding free energies for the ligand pairs 2CQ and 2HCQ, indicating significant differences: 2CQ [2RCQ (−10.44 to −9.93 kcal/mol) and 2SCQ (−9.24 to −8.71 kcal/mol)] and 2HCQ [2RHCQ (−9.66 to −9.31 kcal/mol) and 2SHCQ (−8.76 to −8.21 kcal/mol)]. Furthermore, the Mann–Whitney [32,33] test confirms with 95% confidence that there is a difference in the binding energy distributions (Table S8, Supplementary Information). Taken together, the evidence suggests a slight preference of the β-site for the configured (*R*) enantiomers.

The attractive electrostatic interactions between the ligand and the β-site are crucial in determining the lowest-energy conformations the ligand adopts within the receptor pocket. Furthermore, the susceptibility of enantiomers varies depending on the ionic strength of the solution, suggesting that ionic strength significantly influences ligand binding [11]. Indeed, ionic strength can affect the molecule in hydrated docking by modulating electrostatic interactions, molecular conformation, solvation, and the stability of the complex. By including ions in the simulation, the accuracy of the results can be improved and specific effects, such as the influence of salinity on ligand binding, can be studied.

In this regard, it was considered that since these binding processes take place in an aqueous solution, they should be modulated by solvating water molecules. To evaluate these potential interactions, the solvation entropy at the enzymic pocket with the bound ligand must be considered [34]; thus, a flexible molecular docking analysis with the explicit inclusion of water molecules was performed. In particular, the protocol developed by A. Olson, which calculates water molecules at specific positions on the surface of ligands prior to coupling, was implemented [35,36]. The derived binding score values showed an improvement due to the increase in the solvation potential. In addition, exploring hydration patterns for the protein as a function of the coupled ligand allowed for optimizing the ligand/receptor fit at the binding site.

**Figure 7:**
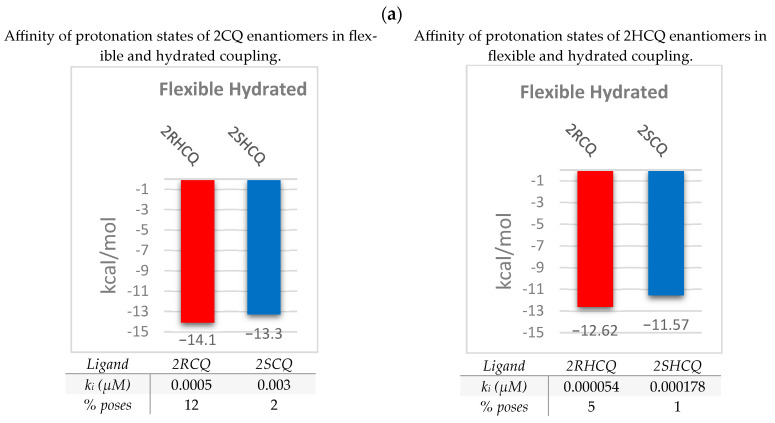

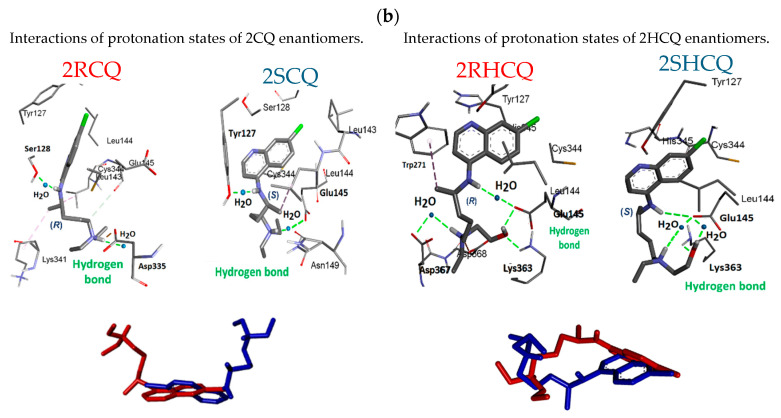
Results of flexible and hydrated coupling: (**a**) Binding affinity of the species protonated at the N-tertiary amino nitrogen of 2RCQ, 2SCQ, 2RHCQ, and 2SHCQ. (**b**) Salient interactions at the ligand–protein complexes and superposition of the enantiomers of 2CQ and 2HCQ (red enantiomer *R* and blue enantiomer *S*). Calculated inhibition constants, ki, in micro molar units, µM, are placed in the shadow data.

The results of the flexible and hydrated docking models with protonated species of 2RCQ, 2SCQ, 2RHCQ, and 2SHCQ are summarized in Figure 7. During the flexible docking simulation, the ligand experienced rotational motions of its single bonds and significant conformational changes due to the flexibility allowed in the enzyme and the presence of pre-calculated water molecules. Figure S1 of the Supporting Information shows the conformational difference before and after docking, illustrating the adaptability of the ligand in the binding site. As expected, water binding occurred mainly in polar areas near the protonated N tertiary amino group and the protonated aniline-type secondary amino group on the ligands.

The resulting hydrated species did participate in hydrogen-bonding interactions with hydrophilic residues within the β-site. For instance, species 2RCQ is involved in hydrogen bond formation that incorporates bridging water between Ser-128 and Asp-335 residues, affording an extended conformation of the ligand. An energy stabilization value of 12.62 kcal/mol, ki = 500 pM, and 12% of the number of poses was estimated. On the other hand, 2SCQ presented two water bridges, one between Tyr-127 and the protonated secondary nitrogen and another between Glu-145 and the protonated tertiary ammonium nitrogen, giving rise to an *extended conformation* and an energy stabilization value of −11.48 kcal/mol, k_i_ = 3000 pM, and 2% number of poses (see Figure 7b, where the blue spheres correspond to water molecules). These results indicate that (*R*) *configured* species bind more efficiently to the β-site.

In the case of the enantiomeric pair of 2HCQ, the hydrogen bonding interactions between the residues of Glu-145 and Asp-367 and the protonated secondary and tertiary amino groups are reflected in a stronger and more negative binding energy (greater stability) relative to the CQ ligands. Figure 7b highlights the water bridges between 2RHCQ and 2SHCQ (hydroxyl and N-tertiary protonated group) and the amino acid residue Glu-145. The calculated binding energy value of −14.1 kcal/mol and −13.3 kcal/mol for 2RHCQ and 2SHCQ, respectively, indicates a more negative (stronger) binding free energy for the (*R*)-*configured* enantiomer (k_i_ = 54 pM and 5% number of poses). The F-tests were unreliable because of insufficient binding free energy data available within each cluster at RMSD = 2 Å.

Likewise, Figure 7b includes the superposition of the enantiomers, revealing significant spatial differences relative to the calculated highest-energy conformations. This allowed us to better understand the changes in the β-site and the enantiomer specificity, which is crucial for understanding the interaction between the ligands and the protein.

Furthermore, we performed simultaneous docking on the whole ACE2 protein using Autodock Vina 1.2.0 software and the multiligand simultaneous docking (MLSD) protocol [37] to evaluate the racemate effect of the ligands (*R*)-2CQ, (*S*)-2CQ, (*R*)-2HCQ, and (*S*)-2HCQ. This approach allowed us to assess the affinity and selectivity of the binding sites on ACE2. Our results reveal that both enantiomers preferentially bind to the α-site, even when ligand pairs with the same configuration (*R*, *R* and *S*, *S*) were tested. This is consistent with the larger size of the α-site (SASA = 304.45 Å^2^). However, we observed a notable difference in the orientation of the (*R*)-enantiomers located closer to the β-site, suggesting higher compatibility and selectivity for this site. The selectivity of binding sites is crucial for understanding protein–ligand interactions. Our results indicate that the α site has a higher affinity for both enantiomers. In contrast, the β-site shows greater selectivity for the (*R*) enantiomer (see Figures S2 and S3 in the Supporting Information).

**Figure 8:**
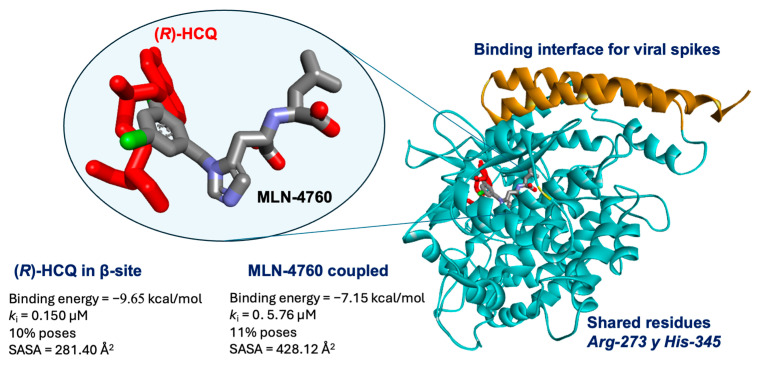
Localization of RHCQ ligand at the β site and MLN-4760 within ACE2, as well as the characteristics of each complex obtained by docking: binding free-energy score (ΔG_Bind_), inhibition constant (k_i_) of the coupled complex, number of poses (%) calculated, and solvent-accessible surface area at the bonding site (SASA).

Finally, the natural ACE2 inhibitor (MLN-4760) redocking was performed to validate our molecular docking studies. As it turns out, the MLN-4760 inhibitor shares similar structural characteristics with HCQ and CQ; for instance, its structure presents several degrees of freedom due to the single bonds within the structure. A RMSD of 0.74 Å was obtained with an affinity energy of −7.15 kcal/mol, k_i_ = 5.76 µM, and 11% number of poses (see Figure S4), and these data demonstrate that the method employed has the capacity to perform accurate molecular docking, with precision similar to that achieved from crystallized structures. Furthermore, Figure 8 shows the overlay of the experimental MLN-4760 structure of the 1R4L protein adjacent to the (*R*)-hydroxychloroquine. Both ligands are very close to the assigned β-site in this work, which leads to the conclusion that the β binding site shares characteristics with the receptor site of the crystallized ligand, which presents similar residues: Arg-273 and His-345. It is also observed that (*R*)-HCQ should be a more efficient inhibitor than (*S*)-HCQ as a result of better binding free energy (−9.65 kcal/mol), a lower inhibition constant (0.150 µM), and a higher percentage of available good poses (10%).

Our molecular docking studies identify three major interaction sites (α, β, and γ) on ACE2 that interact with the enantiomers of CQ and HCQ. We observe that the β-site shows preferential selectivity for species with the (*R*) configuration, whereas the α and γ sites exhibit higher affinity for the (*S*) enantiomer of CQ and HCQ. Ligand stereochemistry, protonation states, and cooperativity between binding sites significantly influence binding. These findings offer detailed insight into the molecular interplay underlying the binding of CQ and HCQ enantiomers to ACE2, which may be critical for understanding the efficacy and selectivity.

### 2.3. Molecular Dynamics

The next step in our study is to investigate the β-site selectivity for CQ and HCQ: (*R*)-2CQ, (*S*)-2CQ, (*R*)-2HCQ, and (*S*)-2HCQ enantiomers. To achieve this, we performed molecular dynamics (MD) simulations to evaluate the conformational processes when the ligand and receptor bind and the conformational perturbations that occur during ligand binding to the β-site.

These MD simulations allowed us to predict the relative stability of the protein–ligand complexes and analyze the binding dynamics. In particular, we determined the RMSD of the docked system and the root mean square fluctuation (RMSF) during binding, which provided us with valuable information on the stability and flexibility of the complexes. Furthermore, we studied the influence of hydrogen bonds in the various complexes, which allowed us to better understand the factors contributing to the β-site selectivity for the CQ and HCQ enantiomers.

**Figure 9:**
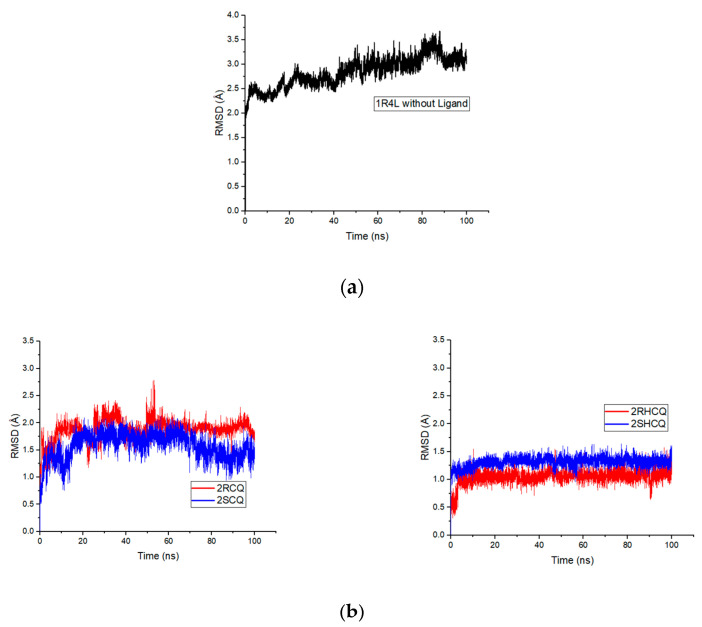

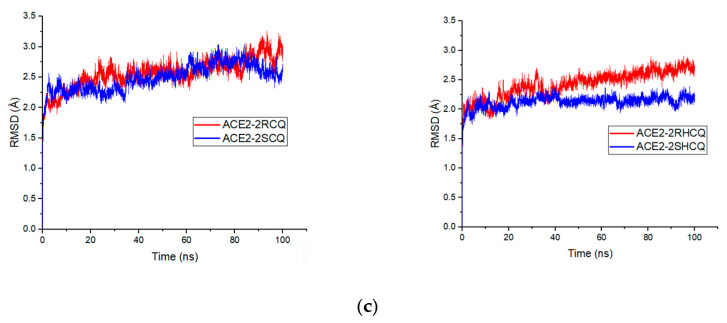
(**a**) RMSD of the 1R4L protein without ligand (control experiment). (**b**) RMSD of the stereoisomers 2RCQ, 2SCQ and 2RHCQ, 2SHCQ (*R* enantiomers are in red and *S* enantiomers are in blue). (**c**) RMSD of the ACE2 protein coupled with each of the stereoisomeric ligands.

In this regard, RMSD refers to measuring structural deviations of the molecular framework (ligand and protein) from its free form to its final conformation after binding. In particular, the structural modifications produced throughout the simulation determine the stability of the protein in terms of its conformation. A stable protein structure shows smaller deviations in the protein’s backbone relative to the free enzyme. In this sense, it is shown that the free protein 1R4L without a ligand shows a positive trend with maximum values of 3.6 Å at 87.8 ns, indicating the destabilization of the protein owing to the absence of the ligand, something that was already anticipated from the control experiment (see Figure 9a). On the other hand, RMSD analysis revealed that with the 2RCQ ligand, the entire coupling ligand trajectory stabilizes after 50 ns, while 2SCQ stabilizes after 75 ns. This indicates that chloroquine’s (*R*) enantiomer adapts more rapidly to the active pocket in ACE2. On the other hand, in the case of the enantiomeric pair in hydroxychloroquine 2RHCQ/2SHCQ, the process reached stabilization after only 20 ns, suggesting that hydrogen bonding involving the hydroxyl group lowers the activation energy for binding. Furthermore, HCQ ligands display fewer structural alterations during binding than their original conformation (Figure 9b). On the other hand, the (*R*) enantiomer of chloroquine 2RCQ presented a positive slope in the RMSD values during the binding process, which implies greater molecular deformation after 50 ns, reaching a maximum deformation value of 2.83 Å at 95 ns. The (*S*) enantiomer 2SCQ exhibited similar behavior with a deviation value of 2.72 Å at 77 ns, which fell to 2.31 Å in the more stable arrangement. A positive slope was observed for 2RHCQ with a maximum deviation of 2.7 Å at 78.6 ns, while 2SHCQ remained stable throughout the simulation (Figure 9c).

Our results suggest that the protein system maintains its original conformation after ligand binding, indicating that ligand incorporation is less disruptive in HCQ-related complexes. Furthermore, none of the ligands caused a significant conformational change in the ACE2 enzyme (average RMSD of the docked protein: 2.40 ± 0.16 Å), suggesting that the open (holoprotein) conformation is not adopted. This implies that CQ and HCQ ligands can effectively prevent viral infection. During molecular dynamics, the ligands underwent conformational changes due to single-bond rotation, but their stereochemistry remained intact and they remained in the β-site. The stability of the complex appears to be determined by spatial and polar–electrostatic complementarity, as well as by hydrophobic contributions that stabilize the binding between the ligand and the protein binding site. The included molecular dynamics video illustrates the stability of the complex and supports this interpretation (See the link at the end of the Supplementary Information).

**Figure 10:**
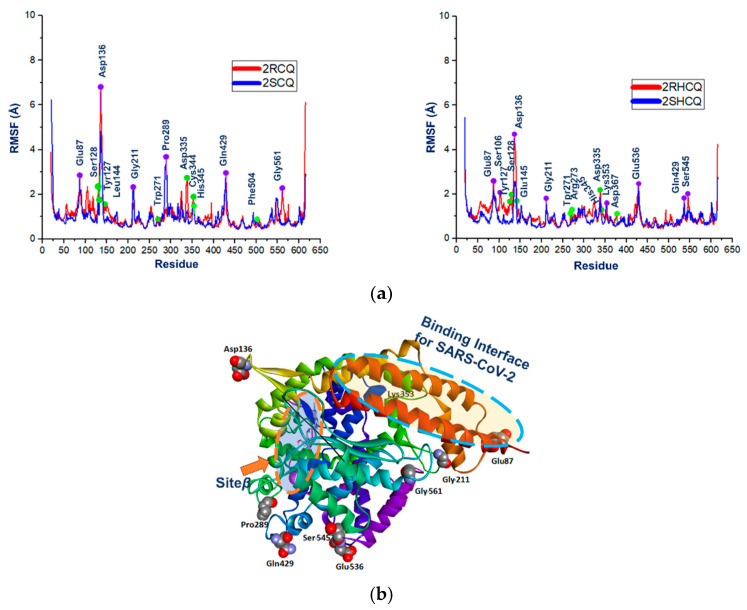
(**a**) RMSF for complexes between ACE2 and stereoisomers of 2CQ/2HCQ. Green points indicate residues of β-site, while violet points indicate other residues. (**b**) Localization of residues within ACE2 fluctuated the most during MD and was found distal to the β-site and viral binding site.

The root-mean-square fluctuation (RMSF) analysis addresses conformational changes during ligand binding in the flexible regions of the protein–ligand complexes. In proteins, a higher RMSF is exhibited by flexible, poorly organized structures such as loops, turns, and coils, while well-structured segments such as those involved in α-helix and β-sheets present slight fluctuations. We have calculated the RMSF value to predict the structural changes induced in the structure of proteins by ligand binding. A small but significant difference in the RMSF was observed between the enantiomeric ligands concerning the fluctuations in the interaction pocket, showing greater fluctuations for the (*R*)-*configured* species 2RCQ and 2RHCQ (see Figure 10a; the interacting amino acid residues at the β-site are shown in green). Regarding the protein framework, greater fluctuations were observed in residues located far from the β-site and the DP domain (binding interface for SARS-CoV-2), particularly in residues that are located in the more flexible regions where the loops, turns, and coils are localized: Glu-87, Asp-136, Gly-211, Pro-289, Lys-353, Gln-429, Glu-536, Ser-545, and Gly-561 (Figure 10a, the participant amino acid residues are highlighted in purple). Figure 10b shows the specific localization of those participant amino acid residues in the protein framework.

Hydrogen bonding interactions play an essential role in stabilizing protein–ligand complexes. In the present study, different strengths of hydrogen bonding interactions among enantiomeric ligands align with distinct affinities between enantiomers towards the β-site. A statistical analysis was performed according to the number of binding events (% frequency) and their distribution over time, obtaining an average number of hydrogen bonds stabilizing the corresponding complexes. The (*R*)-*configured* enantiomer (2RCQ) presented a frequency of 73.6% for these H-bonding interactions after 30 ns. In comparison, the 2SCQ had a higher frequency of 88.6% before 60 ns, which is in line with a higher number of interactions by hydrogen bonding.

**Figure 11:**
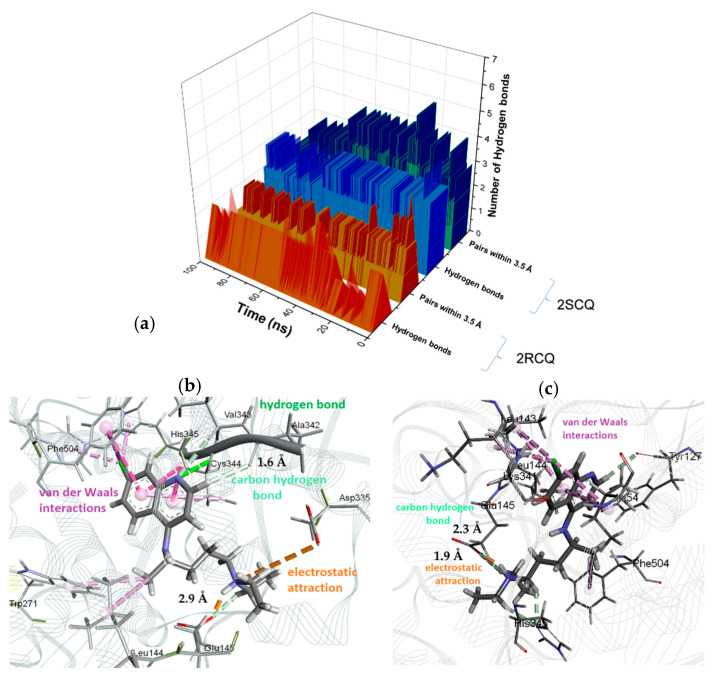
(**a**) Hydrogen bonds between the receptor and enantiomeric 2RCQ and 2SCQ; (**b**) electrostatic interactions (highlighted in red) with 2RCQ; (**c**) van der Waals attractive interactions (highlighted in blue) with 2SCQ.

According to the structural data [38], both enantiomers showed a similar frequency in the participation of H-bonding interactions; in particular, a similar behavior is found in hydrogen bond interactions for the donor–hydrogen–acceptor angle and the distance between the acceptor atom–donor atom. Nevertheless, a lower frequency of 24.7% was observed for the (*R*)-*configured* species relative to the opposite enantiomer (2SCQ, 35.4%), which suggests a higher number of interactions of this type in the latter enantiomer (Figure 11a).

On the other hand, a screenshot of 2RCQ at 30 ns (Figure 11b) shows an extended conformation with a hydrogen bond interaction between Cys-344 and N-aromatic (O-H–N distance = 3.07 Å), together with two simultaneous electrostatic attractive interactions between protonated N-tertiary amine and Glu-145 (NH^+^–O^−^ distance = 1.79 Å) and Asp-335 (HN^+^–O^−^ distance = 5.32 Å). In the case of 2SCQ (time = 55 ns), an extended conformation with an electrostatic attraction interaction between Glu-145 and protonated N-tertiary amine (O^−^–HN^+^ distance = 1.7 Å) is observed, together with weaker hydrogen bonding interactions involving carbon atoms adjacent to the protonated amine as acceptors (Figure 11c).

**Figure 12:**
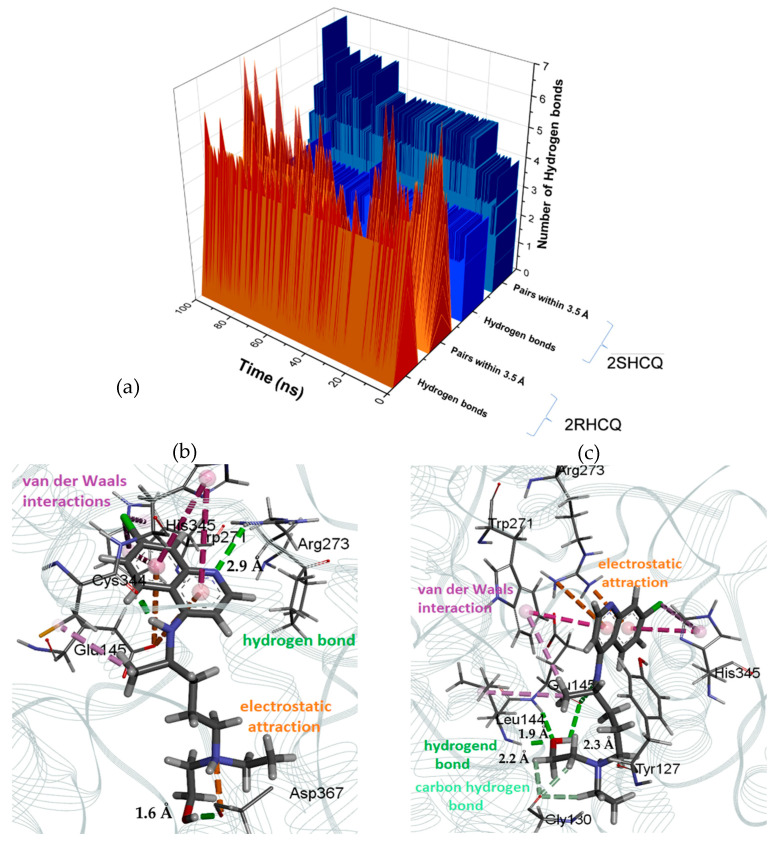
(**a**) Hydrogen bonding interactions involving 2RHCQ and 2SHCQ revealed by MD; (**b**) salient non-bonded interactions with ligand 2RHCQ; (**c**) salient non-bonded interactions with ligand 2SHCQ.

Regarding the 2HCQ enantiomeric pair, the analysis that was carried out according to the geometric criteria for the detection of H-bonding interactions in the ligand complex with the receptor highlights the affinity of the (*R*) enantiomer with a frequency of 61.8% after 30 ns, against 40.42% for the (*S*) enantiomer (Figure 12a). A screenshot at 30 ns shows that 2RHCQ adopts a *bent conformation* with a hydrogen bonding interaction between Asp-367 and the hydroxyl group (O–HO distance = 1.6 Å), along with an electrostatic attractive interaction between Asp-367 and the protonated N-tertiary amino group. An additional non-bonded interaction was observed between Arg-273 and the N-aromatic nitrogen (NH–N^+^ distance = 2.9 Å), as well as two electrostatic attractive interactions with the same residue of Glu-145 and the aromatic system of the ligand (cation–π) (Figure 12b). In contrast, the species 2SHCQ presented two simultaneous hydrogen bonding interactions with the Glu-145 residue and the N-secondary nitrogen (O–HN distance = 2.3 Å), which induced the ligand to adopt a *bent conformation*. This arrangement is reinforced by a simultaneous hydrogen bond interaction between the Leu-144 residue and the ligand’s hydroxyl group (at 2.2 Å and 1.9 Å distances, respectively). At the same time, the aromatic system was anchored by attractive π–π interactions between the aromatic rings of Tpr-271 and His-345 (Figure 12c). These results are consistent with the previous study reported by Machado and [6].

ACE2 displays selectivity for (*R*)-configuration enantiomers at the β-site, resulting in conformational stability of the ligand–ACE2 complexes after 45 ns of simulation. ACE2 has a higher affinity for HCQ than CQ, supporting its potential as a SARS-CoV-2 inhibitor. The (*R*)-enantiomer of HCQ binds more efficiently to ACE2 through electrostatic hydrogen bonding interactions, making it more active. In contrast, CQ’s (*R*)- and (*S*)-enantiomers have similar activity. Ligand binding does not induce significant conformational changes in ACE2.

### 2.4. Free Energy of Binding According to Molecular Mechanics Poisson–Boltzmann Surface Area Solvation Analysis (MM-PBSA)

Molecular mechanics combined with the Poisson–Boltzmann surface area continuum solvation method allowed the estimation of the free energy of the binding of small ligands to biological molecules and was obtained from molecular dynamics simulations of the receptor–ligand complex. In particular, the binding energy reveals how much energy is released when a ligand and protein interact. In this work, the binding energies for each of the four complexes between (*R*)-2CQ, (*S*)-2CQ, (*R*)-2HCQ, and (*S*)-2HCQ and the β-site were estimated separately following a molecular dynamics simulation. The energies that contribute to the total interaction-free energy (van der Waals energy, electrostatic energy, polar solvation energy, and solvent-accessible surface energy (SASA)) were calculated to establish which type of non-bonded interaction has the most significant contribution to the stability of the final complex.

MM-PBSA calculations (Table 3) reveal that both 2CQ and 2HCQ enantiomers exhibit significant affinity for the binding site. For 2CQ, both enantiomers (R and S) show similar total binding free energies (−32.21 ± 6.62 kcal/mol and −33.22 ± 3.91 kcal/mol, respectively), whereas for 2HCQ, the (*R*)-enantiomer exhibits a slightly higher affinity (−39.04 ± 6.09 kcal/mol) compared to the (*S*)-enantiomer (−36.52 ± 4.46 kcal/mol). In both cases, favorable van der Waals and electrostatic interactions compensate for the unfavorable desolvation, allowing binding. Desolvation is primarily due to the hydrophobic nature of the ligand, specifically the aromatic system and the carbon chain, which results in a loss of interactions with water molecules and a reduction in the system’s entropy.

It is important to emphasize that these results must be interpreted within the context of the limitations of the MM-PBSA method. Although they provide a detailed estimate of the binding free energy, they may not fully capture the complexity of the system’s conformational dynamics and the contribution of the receptor’s conformational entropy. Despite these limitations, the results suggest that the enantiomers of 2CQ and 2HCQ have significant affinity for the binding site, which can be useful in guiding ligand design and binding optimization.

Our study has intrinsic limitations arising from the dependence of docking and molecular dynamics methods on force fields. These methods may not fully capture the complexity of molecular interactions, including dynamical and solvation effects, which may influence the accuracy of the results. Additionally, accurate crystallographic data are lacking and the molecular interactions are complex. Nevertheless, our results, based on in silico simulations, provide detailed insights into the interactions between CQ, HCQ, and ACE2. Our findings reveal the discrepancy associated with the enantioselectivity of the drugs, a topic that has not been clearly defined in the literature due to the multitude of ACE2 interaction sites. Our findings suggest that the (*R*) enantiomer of HCQ binds more efficiently to ACE2 through electrostatic interactions, positioning it as a potential SARS-CoV-2 inhibitor.

However, the literature suggests that the (*S*) enantiomer of HCQ may exhibit greater acute toxicity in vivo, an apparent discrepancy between antiviral efficacy and toxicity. This could be due to the complex interaction between HCQ and ACE2, involving multiple binding sites and the allosteric reorganization of the protein. Additionally, the enantioselective pharmacokinetics of HCQ, which are characterized by higher blood concentrations of the (*R*) enantiomer and higher (*S*) enantiomer metabolites, may contribute to the discrepancy between antiviral efficacy and toxicity.

Despite these complexities, our results underscore the importance of considering enantioselectivity when developing drugs against SARS-CoV-2. A deeper understanding of the interaction between hydroxychloroquine (HCQ) and angiotensin-converting enzyme 2 (ACE2), as well as the enantioselective pharmacokinetics and pharmacodynamics of HCQ, is essential for developing safer and more effective drugs. Our findings provide a molecular basis for understanding the differences in the binding and activity of the (*R*) and (*S*) enantiomers of HCQ. This information could be useful for designing new drugs and optimizing their efficacy and safety.

## 3. Materials and Methods

The relative binding ability of each enantiomer of CQ and HCQ was determined according to the following steps: (I) the modeling of the free ligands and the receptor protein, (II) exploration of the pharmacological potential of the ligands and prediction of the protein’s interaction region, (III) molecular docking on ACE2, (IV) simulation of molecular dynamics upon binding, and (V) estimation of binding free energies using the Molecular Mechanics Poisson–Boltzmann Surface Area (MM-PBSA) method.

### 3.1. Ligand’s Modeling

Three-dimensional 3D representations of the drugs of interest were downloaded from the DrugBank (IDs for CQ and HCQ are DB00608 and DB01611, respectively) and were adjusted to afford the corresponding enantiomeric structures with the Avogadro R© 1.2.0 software. The optimization of geometries and electronic charges was achieved by using a functional B3LYP/6-31G and converted to .pdb [39]. With the SPORES program (Protonation and Structure Recognition System), the structures of the protonated states of the ligands were then determined [40].

### 3.2. Protein Modeling

The crystal structure of ACE2, obtained at pH = 7.5 [16] (PDB ID 1R4L), was acquired in .pdb format from the Protein Databank (RCSB PDB) [41] and simulated by removing the original ligand and water molecules, maintaining only the A chain of the receptor, with the PyMOL Molecular Graphics System, Version 2.0 Schrödinger, LLC software. The SPORES program was used to define the protonation states of the protein at pH = 7.4.

### 3.3. Molecular Docking

An initial prediction of the amino acid residues where the CQ/HCQ ligand molecules are likely to bind was made using the available web server Protein-plus (https://proteins.plus/, accessed on 25 June 2025) and subsequently compared with a molecular docking simulation in the whole protein (blind coupling), as well as in the interaction regions reported in the literature. In addition, a prediction of the pharmacological potential (Druggability) of the protein with these ligands was made by DoGSiteScorer, accessible in Protein-plus [20,21].

The coupling processes of the enantiomeric CQ and HCQ ligands on ACE2 were modeled using Autodock vina 1.2.0 [37], the multiligand simultaneous docking was performed with the MSD protocol [37] on the whole protein to evaluate the racemate effect of ligands (R)-2CQ, (S)-2CQ, (R)-2HCQ and (S)-2HCQ, the results where refined with the AutoDockTools and AutoDock [42] softwares. The molecules’ potential protonation states (Figure 3) were considered to explore the most salient interaction regions. The binding free energy score value and the degree of occupation for each conformer in a group, which depends on an RMSD, were established as limiting criteria [43].

Blind coupling: It was initiated with free ligands, adding Gasteiger charges, connecting nonpolar hydrogen bonds, and establishing rotating bonds. A box capable of containing the entire structure was built into the protein. With the AutoGrid program, affinity maps were calculated for all types of atoms present and an electrostatic map with a spacing of 0.53 Å and grid dimensions of 120 × 120 × 120 Å was created. The center of the grid was set at coordinates 37.846, 3.091, and 22.477 Å at x, y, and z, respectively. Gasteiger-type charges were assigned and polar hydrogens were added to the entire receptor. The couplings were made by Autodock 4.2, regulating the parameters of the genetic algorithm (GA) and programming a rigid macromolecule and flexible ligands. In total, 150 GA executions were used, with a population of 150 conformers, and 25,000,000 evaluations were carried out. In the binding sites defined by blind coupling, three types of couplings were modeled: localized coupling, flexible coupling, and flexible/hydrated coupling. Localized coupling was performed by keeping the protein rigid and the ligand flexible. In contrast, the flexible and flexible/hydrated couplings were modeled so that some protein residues (particularly Ser-128, Glu-145, Cys-344, Asp-335, and Asp-367) and the ligand were flexible. Similarly, the most advanced docking (flexible/hydrated) simulations incorporated water molecules pre-calculated by the software (see Olson’s protocol) [32,33]. The conditions are shown in Table S1 of the Supporting Information. The structures were processed with the Discovery Studio Visualizer program, V2.1.

### 3.4. Molecular Dynamics Simulation

Once the initial structural models have been built, the surrounding environment must be considered, as the rigorous mimicking of realistic solvent conditions during simulation is key to producing reliable results. Calculations are carried out at pH 7.2, using programs such as PROPKA [44] to account for the protonation states of the protein. Simulations of the four coupled complexes were developed for 2RCQ, 2SCQ, 2RHCQ, and 2SHCQ (ligands protonated at the amino terminal group), plus that of the unliganded protein (control experiment) using the program GROMACS 2020 version 5 [35], with force field AMBER99SB-ILDN [45] on the CentOS Linux operating system release 7.5.1804. The TIP3P water model was chosen for simulations in explicit solvation. The ACPYPE tool (Antechamber PYthon Parser interface) GNU GPL version 3 created the ligand topology files [46].

A cubic box with the dimensions of 75.74 Å, 61.62 Å, and 67.40 Å was programmed for periodic boundary conditions, maintaining a minimum distance of 10 Å from any atom to the boundary of the box. The system was neutralized by adding sodium (Na^+^) and chloride (Cl^−^) ions at a concentration of 0.15 M. The steepest descent algorithm was applied to eliminate any steric collision or unusual geometry. The equilibrium was carried out in two steps to balance the solvent around the protein–ligand complex: first, the equilibrium of the system about NVT at a temperature of 300 K (100,000 ps), and then the equilibrium of the system in relation to NPT at 1 bar pressure per 100,000 ps. After balancing the system, MD production was run for 100 ns and defined with 50,000,000 steps. Trajectories were analyzed using GROMACS and produced with VMD—Visual Molecular Dynamics—tools and Camtasia Studio Version 8.1.2 (2013). Graphs representing the simulation results were plotted using Origin2021 (Origin Lab Corporation, Northampton, MA, USA).

### 3.5. Binding Free Energy by MMPBSA

For each system, the free binding energy was calculated using the MM-PBSA technique based on the final structure derived from the final stage of production of the molecular dynamics, where ∆GT is decomposed in terms of electrostatic interactions (∆Elec), van der Waals (∆Evdw), and solvation (∆Gsol), containing polar parts and non-polar parts. With the Python extension ante-MMPBSA.py [47], it was possible to generate the individual solvated and unsolvated topology files for the ligand and the protein, checking them before performing the corresponding energy calculations. The surface tension constant was taken as 0.02267 kJ mol^−1^ Å^−2^ and the SASA constant as 3.84928 kJ/mol for adjustment [48].

## 4. Conclusions

The present study elucidates the molecular interaction between CQ and HCQ with the human ACE2 enzyme. Three major interaction sites (α, β, and γ) with specific preferences were identified. The α and γ sites exhibit a preference for (*S*) enantiomers, while the higher-affinity β site demonstrates a clear preference for (*R*) enantiomers, accompanied by specific electrostatic interactions.

Several factors, including ligand stereochemistry, protonation states, and the combined activity of the binding sites, influence the binding process. ACE2 exhibits a higher affinity for HCQ than for CQ, thus supporting its potential as a SARS-CoV-2 inhibitor. The (*R*)-*enantiomer* of HCQ has been shown to bind more efficiently to ACE2 through electrostatic hydrogen bonding interactions, resulting in increased activity. The results obtained demonstrate the complexity of drug binding to ACE2 and underscore the significance of considering the interplay between binding sites and protonation states. It is acknowledged that HCQ, particularly the (*R*)-*enantiomer*, has the potential to serve as an effective inhibitor of SARS-CoV-2. However, concerns regarding its toxicity have been raised, underscoring the necessity for a more profound comprehension of the molecular mechanism through which these drugs bind to the ACE2 receptor. This enhanced understanding is pivotal for the development of efficacious and safe therapeutic strategies.

## Figures and Tables

**Table 1:** Comparison of the main interactions between binding sites (α, β, and γ), (*R*) and (*S*) enantiomers of CQ, and the corresponding protonated species.

Site	α		γ		β	
^a^ SASA (A^2^)	304.45		299.90		281.40	
^b^ Ligand	RCQ	SCQ	RCQ	SCQ	RCQ	SCQ
Binding energy (kcal/mol)	−5.7	−6.42	−8.08	−8.01	−9.45	−8.37
^c^ k_i_ (µM)	66.88	19.62	1.2	1.34	0.152	0.237
^d^ Poses (%)	23	25	15	21	8	3

^a^ Solvent-accessible surface area (SASA) at the binding site. ^b^ Ligand (*R* or *S* descriptor indicates the configuration of the ligand). ^c^ Inhibition constant (k_i_) of the coupled complex. ^d^ Number of poses (%): grouping of structures of docked conformations determined by the RMSD tolerance specified in 2 Å.

**Table 2:** Comparison of the main interactions between binding sites (α, β, and γ), (*R*) and (*S*) enantiomers of HCQ, and the corresponding protonated species.

Site	α		γ		β	
^a^ SASA (A^2^)	304.45		299.90		281.40	
^b^ Ligand	RHCQ	SHCQ	RHCQ	SHCQ	RHCQ	SHCQ
Binding energy (kcal/mol)	−9.24	−10.33	−7.78	−8.38	−9.65	−8.28
^c^ k_i_ (µM)	169.61	126.75	1.99	0.720	0.150	0.846
^d^ Number of poses (%)	17	19	17	21	10	2

^a^ Solvent-accessible surface area (SASA) at the binding site. ^b^ Ligand (*R* or *S* descriptor indicates the configuration of the ligand). ^c^ Inhibition constant (k_i_) of the coupled complex. ^d^ % Number of poses: storing of docked conformations structures determined by the RMSD tolerance specified in 2 Å.

**Table 3:** Decomposed non-bonded interaction energies between the enantiomeric pairs of 2CQ and 2HCQ with ACE2 and evaluated standard deviations of the MM-PBSA analysis (all energies are in kcal/mol).

Ligand	van der Waals Energy	Electrostatic Energy	Polar Solvation Energy	Nonpolar Solvation Energy, SASA	Total Binding Free Energy
2RCQ	−43.84 ± 3.12	−24.31 ± 4.09	45.01 ± 2.44	−4.29 ± 0.85	−32.21 ± 6.62
2SCQ	−44.28 ± 2.39	−27.64 ± 5.27	42.06 ± 3.19	−2.76 ± 2.33	−33.22 ± 3.91
2RHCQ	−45.15 ± 3.84	−34.73 ± 6.28	52.46 ± 2.96	−4.42 ± 0.28	−39.04 ± 6.09
2SHCQ	−44.63 ± 3.81	−32.66 ± 5.21	48.64 ± 3.90	−4.17 ± 1.69	−36.52 ± 4.46

## Data Availability

The original contributions presented in this study are included in the article/supplementary material. Further inquiries can be directed to the corresponding authors.

## Supplementary Materials

The following supporting information can be downloaded at: https://www.mdpi.com/article/10.3390/ph18070982/s1, Figure S1: Conformational difference showing the conformational difference before and after flexible and hydrated docking illustrating the adaptability of the ligand in the binding site; Figure S2: Multiple Ligand Simultaneous Docking (MLSD). Both enantiomers preferentially bound to the α site due to their larger size; Figure S3: Multiple Ligand Simultaneous Docking (MLSD); Figure S4: Molecular recoupling of the ligand MLN-4760 on crystal structure of ACE2 (PDB ID: 1R4L); Table S1: The cluster energy of the docked conformers is determined by the RMSD tolerance specified in 2Å (2RCQ Vs. 2SCQ and 5RCQ Vs. 5SCQ); Table S2: F-Test Two-Sample for Variances (2RCQ Vs. 2SCQ); Table S3: F-Test Two-Sample for Variances (5RCQ Vs. 5SCQ); Table S4: The cluster energy of the docked conformers is determined by the RMSD tolerance specified in 2Å (2RHCQ Vs. 2SHCQ and 5RHCQ Vs. 5SHCQ); Table S5: F-Test Two-Sample for Variances (2RHCQ Vs. 2SHCQ); Table S6: F-Test Two-Sample for Variances (5RHCQ Vs. 5SHCQ); Table S7: Mann Whitney test for 2CQ and 2HCQ; Table S8: Molecular coupling execution conditions.
