# Supplementary material for: A New In Silico Comparison of the Relative Affinity of Enantiomeric Chloroquine (CQ) and Hydroxychloroquine (HCQ) for ACE2
Source: Pharmaceuticals (Basel). 2025 Jun 30;18(7):982. doi: 10.3390/ph18070982 (PMC12299195; doi:10.3390/ph18070982)
Supplement: Supplementary file 1 [file pharmaceuticals-18-00982-s001.zip › pharmaceuticals-3578945-supplementary.pdf]

Supporting Information

A new *in silico* comparison of the relative affinity of enantiomeric chloroquine (CQ) and hydroxychloroquine (HCQ) for ACE2

Carlos Naranjo-Castañeda[1], Marco A. García-Revilla,*[,2] Eusebio Juaristi*[,1,3]

[1]Department of Chemistry, Centro de Investigación y de Estudios Avanzados del Instituto Politécnico Nacional, Avenida Instituto Politécnico Nacional No. 2508, San Pedro Zacatenco, 07360 Mexico City, Mexico.

E-mail: ejuarist@cinvestav.mx

[2]Department of Chemistry, Universidad de Guanajuato, Noria Alta S/N, 36050 Guanajuato, Mexico.

E-mail: magarcia@ugto.mx

[3]El Colegio Nacional, Donceles # 104, Centro Histórico, 06020 Mexico City, Mexico.

From Figure 6, we have performed the F-test (with parameter alpha equal to 0.05) and determined with a confidence bar of 95% level to determine whether there is a significant difference in the variation of the reported results obtained from the molecular coupling comparing the relative affinity of enantiomeric pairs. In addition, this study proposes a Mann-Whitney-type comparison test to compare the distributions of the free energy of the pairs of enantiomers with 95% confidence.

F test

The test was performed using Microsoft 365 Excel.

Table S1. The cluster energy of the docked conformers is determined by the RMSD tolerance specified in 2Å.

2RCQ		2SCQ		5RCQ		5SCQ	
Binding energy (kcal/mol)	inhibition constant (μM)	Binding energy (kcal/mol)	inhibition constant (μM)	Binding energy (kcal/mol)	inhibition constant (μM)	Binding energy (kcal/mol)	inhibition constant (μM)
-11.32	0.005	-10.16	0.014	-8.32	0.795	-8.25	0.898
-11.15	0.009	-10.09	0.035	-8.15	1.24	-8.21	0.951
-11.01	0.012	-9.74	0.070	-8.13	1.41	-8.1	1.15
-10.92	0.018	-9.45	0.122	-7.9	1.57	-7.66	2.42
-10.85	0.026	-9.26	0.176	-7.86	1.71	-7.63	2.56
-10.74	0.035	-9.08	0.219	-7.23	2.18	-7.62	2.71
-10.65	0.042	-8.62	0.406	-6.78	12.09	-7.6	2.73
-10.5	0.045	-8.35	0.702			-7.58	2.76
-10.42	0.048	-8.03	0.918			-7.51	3.35
-10.34	0.053					-7.23	3.39
-10.3	0.055					-6.9	36.91
-10.23	0.058						
-10.2	0.060						
-10.1	0.062						
-9.96	0.065						
-9.95	0.068						
-9.93	0.077						
-9.86	0.080						
-9.85	0.085						
-9.82	0.092						
-9.35	0.108						
-9.23	0.120						
-8.92	0.129						
-8.89	0.315						

Hypothesis test

Null hypothesis
$H_0: \sigma^2_{2RCQ} = \sigma^2_{2SCQ}$
The variances are equal

Alternative Hypothesis
$H_1: \sigma^2_{2RCQ} \neq \sigma^2_{2SCQ}$
The variances are different

Table S2. F-Test Two-Sample for Variances (2RCQ Vs. 2SCQ)

	2RCQ	***2SCQ***
Mean ($\bar{x}$)	-10.19	-9.20
Standard deviation (s)	0.66	0.75
Standard error (SE)	0.13	0.25
Variance (s^2)	0.44	0.56
Observations	24	9
Degrees of freedom	23	8
F_statistic (F)	1.29	
F Critical (alpha 0.05)	2.37	

$$F\_statistic = \frac{s^2_{2SCQ}}{s^2_{2RCQ}} = 1.29$$

F < $F_{critical}$ therefore the null hypothesis is accepted. The variances are equal between. the ligands.

2RCQ
The confidence interval (CI) 95% Z-Score = 1.96
$CI_{95\%\,X} = \bar{x} \pm 1.96\,SE$
$CI_{95\%\,X} = -10.19 \pm 1.96\ (0.13)$
$CI_{95\%\,X} = [-10.44\ to\ -9.93]$

2SCQ
The confidence interval (CI) 95% Z-Score = 1.96
$CI_{95\%\,X} = \bar{x} \pm 1.96\,SE$
$CI_{95\%\,X} = -9.20 \pm 1.96\ (0.25)$
$CI_{95\%\,X} = [-9.24\ to\ -8.71]$

Hypothesis test

Null hypothesis
$H_0: \sigma^2_{5RCQ} = \sigma^2_{5SCQ}$
The variances are equal

Alternative Hypothesis
$H_1: \sigma^2_{5RCQ} \neq \sigma^2_{5SCQ}$
The variances are different

Table S3. F-Test Two-Sample for Variances (5RCQ Vs. 5SCQ)

	5RCQ	***5SCQ***
Mean ($\bar{x}$)	-7.77	-7.66
Standard deviation (s)	0.56	0.41
Standard error (SE)	0.21	0.12
Variance (s^2)	0.31	0.16
Observations	7	11
Degrees of freedom	6	10
F_statistic (F)	1.90	
F Critical (alpha 0.05)	3.21	

$$F\_statistic = \frac{s^2_{5RCQ}}{s^2_{5SCQ}} = 1.90$$

F < $F_{critical}$ therefore the null hypothesis is accepted. The variances are equal between the ligands.

5RCQ
The confidence interval (CI) 95% Z-Score = 1.96
$CI_{95\%\,X} = \bar{x} \pm 1.96\,SE$
$CI_{95\%\,X} = -7.77 \pm 1.96\ (0.21)$
$CI_{95\%\,X} = [-8.18\ to\ -7.35]$

5SCQ
The confidence interval (CI) 95% Z-Score = 1.96
$CI_{95\%\,X} = \bar{x} \pm 1.96\,SE$
$CI_{95\%\,X} = -7.66 \pm 1.96\ (0.12)$
$CI_{95\%\,X} = [-7.89\ to\ -7.42]$

Table S4. The cluster energy of the docked conformers is determined by the RMSD tolerance specified in 2Å.

2RHCQ		***2SHCQ***		***5RHCQ***		***5SHCQ***	
Binding energy (kcal/mol)	inhibition constant (μM)	Binding energy (kcal/mol)	inhibition constant (μM)	Binding energy (kcal/mol)	inhibition constant (μM)	Binding energy (kcal/mol)	inhibition constant (μM)
-10.43	0.022	-9.23	0.171	-7.23	2.35	-8.19	0. 991
-10.28	0.031	-9.16	0.198	-7.15	5.04	-7.92	1.41
-10.25	0.033	-9.15	0.234	-7.08	6.57	-7.82	2.11
-10.22	0.037	-9.14	0.278			-7.76	3.47
-10.01	0.043	-9.13	0.335			-7.6	3.61
-9.96	0.050	-9.09	0.634			-7.54	4.2
-9.95	0.054	-8.96	0.832				
-9.89	0.058	-8.77	1.15				
-9.85	0.063	-8.51	1.43				
-9.74	0.068	-8.36	1.73				
-9.64	0.089	-8.27	1.85				
-9.57	0.093	-8.21	2.09				
-9.54	0.099	-7.99	2.35				
-9.45	0.143	-7.92	2.66				
-9.32	0.187	-7.87	3.17				
-9.27	0.196	-7.86	3.33				
-9.26	0.203	-7.58	3.82				
-9.25	0.219	-7.55	4.44				
-9.24	0.223						
-9.23	0.239						
-9.23	0.243						
-9.19	0.257						
-9.16	0.263						
-9.15	0.271						
-9.14	0.280						
-8.89	0.285						
-8.76	0.319						
-8.72	0.443						
-8.52	0.724						

Hypothesis test

Null hypothesis	Alternative Hypothesis
$H_0: \sigma^2_{2RHCQ} = \sigma^2_{2SHCQ}$	$H_1: \sigma^2_{2RHCQ} \neq \sigma^2_{2SHCQ}$
The variances are equal	The variances are different

Table S5. F-Test Two-Sample for Variances (2RHCQ Vs. 2SHCQ)

	2RHCQ	***2SHCQ***
Mean (x̅)	-9.49	-8.49
Standard deviation (s)	0.50	0.60
Standard error (SE)	0.09	0.14
Variance (s^2)	0.25	0.36
Observations	29	18
Degrees of freedom	28	17
F_statistic (F)	1.44	
F Critical (alpha 0.05)	2.02	

$$F\_statistic = \frac{S^2_{2SHCQ}}{S^2_{2RHCQ}} = 1.44$$

F < $F_{critical}$ therefore the null hypothesis is accepted. The variances are equal between the ligands.

2RHCQ
The confidence interval (CI) 95% Z-Score = 1.96
$$CI_{95\%\,X} = \bar{x} \pm 1.96\,SE$$
$$CI_{95\%\,X} = -9.49 \pm 1.96\ (0.09)$$
$$CI_{95\%\,X} = [-9.66\ to\ -9.31]$$

2RHCQ
The confidence interval (CI) 95% Z-Score = 1.96
$$CI_{95\%\,X} = \bar{x} \pm 1.96\,SE$$
$$CI_{95\%\,X} = -8.49 \pm 1.96\ (0.14)$$
$$CI_{95\%\,X} = [-8.76\ to\ -8.21]$$

Hypothesis test

Null hypothesis	Alternative Hypothesis
$H_0: \sigma^2_{5RHCQ} = \sigma^2_{5SHCQ}$	$H_1: \sigma^2_{5RHCQ} \neq \sigma^2_{5SHCQ}$
The variances are equal	The variances are different

Table S6. F-Test Two-Sample for Variances (5RHCQ Vs. 5SHCQ)

	5RHCQ	***5SHCQ***
Mean (x̅)	-7.15	-7.81
Standard deviation (s)	0.08	0.23
Standard error (SE)	0.04	0.10
Variance (s^2)	0.01	0.06
Observations	3	6
Degrees of freedom	2	5
F_statistic (F)	0.16	
F Critical (alpha 0.05)	5.78	

$$F\_statistic = \frac{S^2_{5SHCQ}}{S^2_{5RHCQ}} = 0.16$$

F < $F_{critical}$ critical therefore the null hypothesis is accepted. The variances are equal between the ligands.

5RHCQ
The confidence interval (CI) 95% Z-Score = 1.96
$$CI_{95\%\,X} = \bar{x} \pm 1.96\,SE$$
$$CI_{95\%\,X} = -7.15 \pm 1.96\ (0.04)$$
$$CI_{95\%\,X} = [-7.22\ to\ -7.07]$$

5RHCQ
The confidence interval (CI) 95% Z-Score = 1.96
$$CI_{95\%\,X} = \bar{x} \pm 1.96\,SE$$
$$CI_{95\%\,X} = -7.81 \pm 1.96\ (0.10)$$
$$CI_{95\%\,X} = [-8.00\ to\ -7.61]$$

Mann Whitney test

Table S7. Mann Whitney test for 2CQ and 2HCQ

2RCQ Vs. 2SCQ		2RHCQ Vs. 2SHCQ	
P value	0.0019	P value	<0.0001
Exact or approximate P value?	Exact	Exact or approximate P value?	Exact
P value summary		P value summary	
Significantly different (P < 0.05)?	Yes	Significantly different (P < 0.05)?	Yes
One- or two-tailed P value ?	Two-tailed	One- or two-tailed P value?	Two-tailed
Sum of ranks in column 2RCQ, 2SCQ	334, 227	Sum of ranks in column 2RHCQ, 2SHCQ	475.5, 652.5
Mann-Whitney U	34	Mann-Whitney U	40.5
Difference between medians		Difference between medians	
Median of 2RCQ	-10.22, n=24	Median of 2RHCQ	-9.320, n=29
Median of 2SCQ	-9.260, n=9	Median of 2SHCQ	-8.435, n=18
Difference: Actual	0.955	Difference: Actual	0.885
Difference: Hodges-Lehmann	0.955	Difference: Hodges-Lehmann	0.99

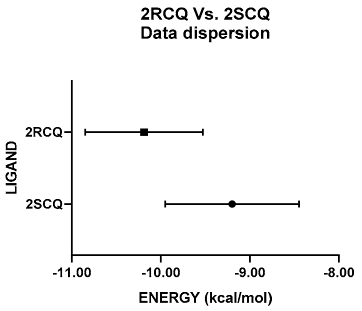

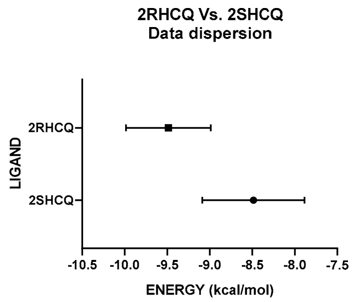

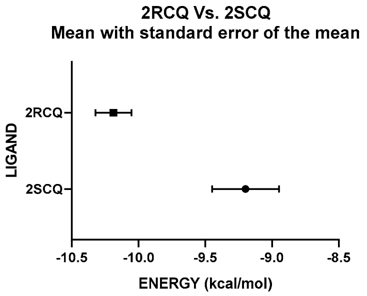

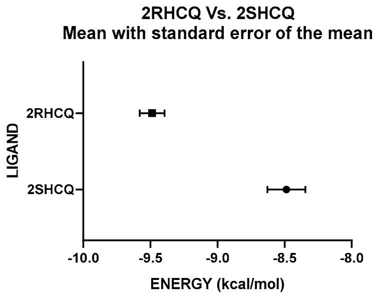

For the F test, we found that the null hypothesis is accepted for all cases, indicating that the dispersion of the data within each group around the mean is similar. However, we must take this information with caution because of the discrepancy in the binding free energy sample sizes and the fact that the data from the docking experiment does not necessarily show a normal distribution. To support the results of the F test with the standard error bar we determined that there is no overlap between the energy intervals for the ligand pairs 2CQ [2RCQ (−10.44 to −9.93 kcal/mol) and 2SCQ (−9.24 to −8.71 kcal/mol)] and 2HCQ [2RHCQ (9.66 to −9.31kcal/mol) and 2SHCQ (−8.76 to −8.21kcal/mol)]. (with a confidence level of 95% for each enantiomer). On the other hand, the non-parameterized

Mann-Whitney test (95% confidence) shows that the distributions of the binding free energy are different. This evidence demonstrates a slight preference for the *β* site for the configured (*R*) enantiomers.

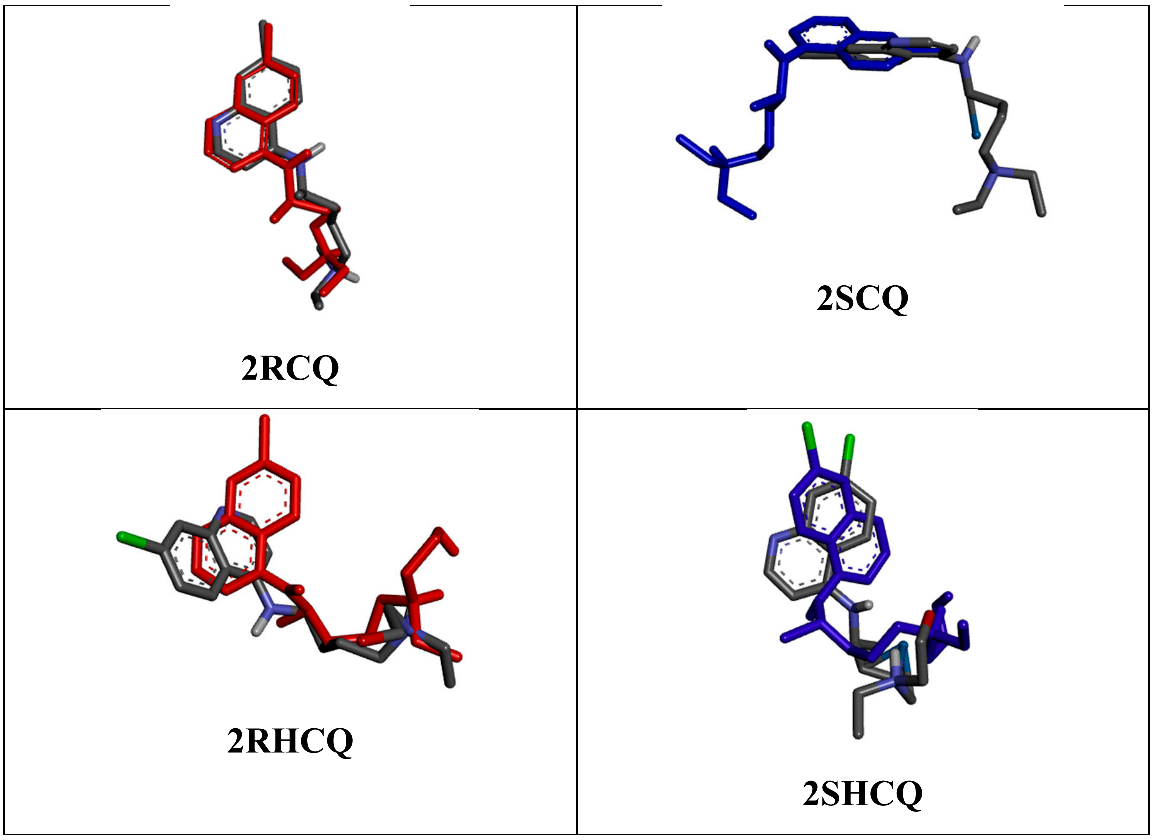

Figure S1. Conformational difference showing the conformational difference before and after flexible and hydrated docking illustrating the adaptability of the ligand in the binding site. Red structures initial structure, gray structures final structure.

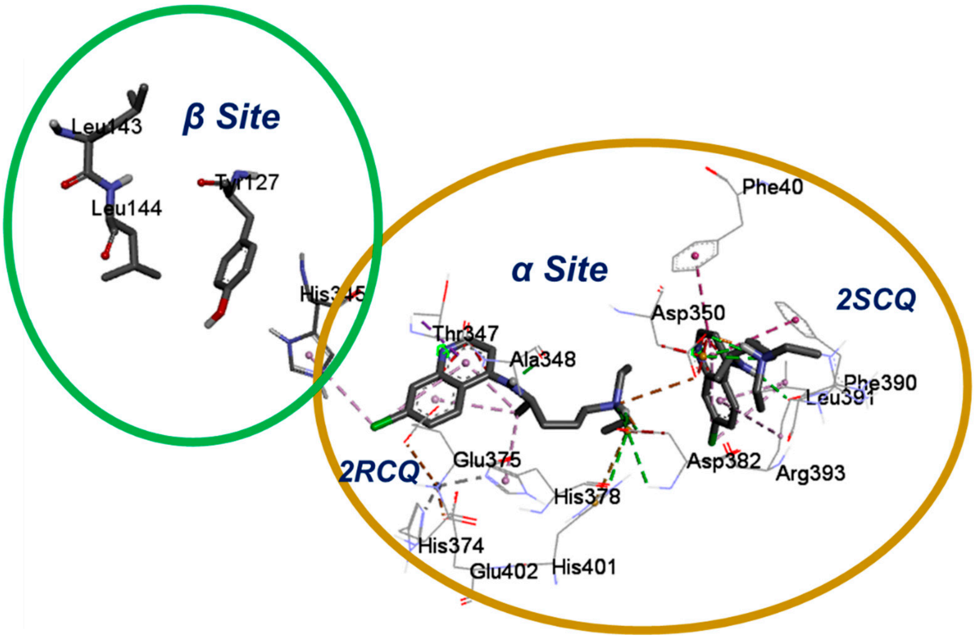

Figure S2. Multiple Ligand Simultaneous Docking (MLSD). Both enantiomers preferentially bound to the α site due to their larger size. The R enantiomer (2RCQ) showed greater affinity for the β site, orienting itself closer to this site.

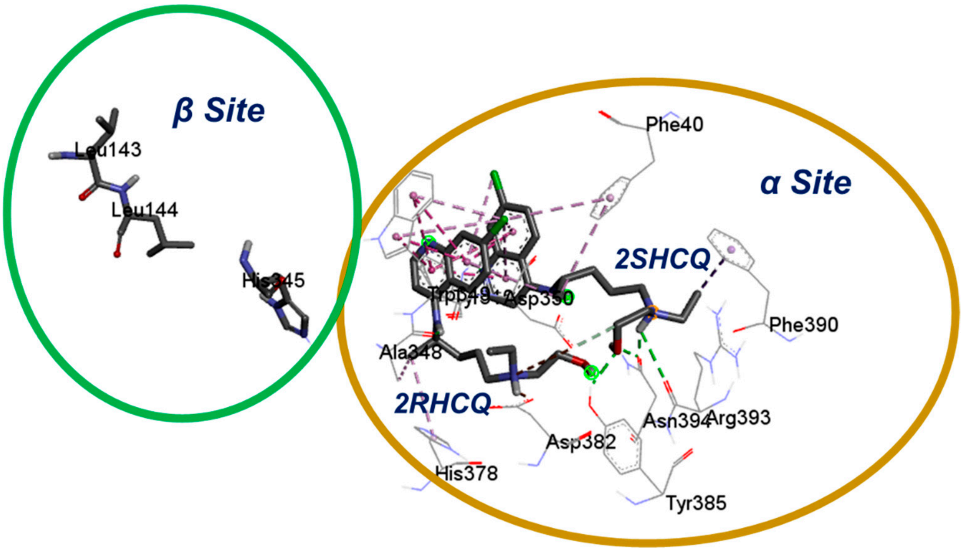

Figure S3. Multiple Ligand Simultaneous Docking (MLSD). Both enantiomers preferentially bound to the α site due to their larger size. The R enantiomer (2RCQ) showed greater affinity for the β site, orienting itself closer to this site.

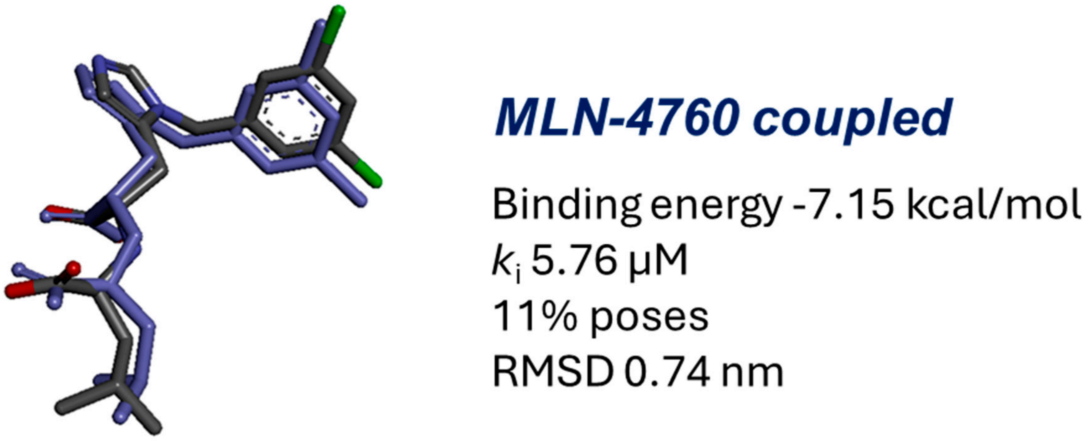

Figure S4. Molecular recoupling of the ligand MLN-4760 on crystal structure of ACE2 (PDB ID: 1R4L). The original structure of the crystal is shown in violet.

Table S8. Molecular coupling execution conditions

	site α			site β			site γ		
	Rigid	Flexible	Flexible/ Hydrated	Rigid	Flexible	Flexible/ Hydrated	Rigid	Flexible	Flexible/ Hydrated
**Grid spacing (Å)**	0.375			0.375			0.375		
**Box size ($Å^3$)**	70*48*56	50*38*42		60*40*80	50*50*46		42*40*64	48*50*48	
**Coordinates of Central Grid Point of Maps**	28.309, 14.972, 21.108			39.902, 3.091,35.452	39.902, 3.091,35.452		23.747,5.152, 12.60	25.95, 6.444, 8.771	
**Algorithm**	genetic algorithm (GA)			Genetic algorithm (GA)			Genetic algorithm (GA)		
**Number of GA runs**	200	300	150	200	300	150	200	300	150
**Population size**	150			150			150		
**Maximum number of evaluations**	25 x 10^6			25 x 10^6			25 x 10^6		

Molecular Dynamic Videos of the studied systems are available at:

https://cinvestav365-my.sharepoint.com/:f:/r/personal/carlos_naranjo_cinvestav_mx/Documents/movies%20MD?csf=1&web=1&e=9Ji7R9&xsdata=MDV8MDJ8bWFnYXJjaWFAdWd0by5teHxkYjY1NjMwNTExNjM0ZjYxYThkYjA4ZGQwNDAxNjk5ZnwxMzJiOTg3MWUwMjU0ZWFkYTM0ZDdiZDVlN2EzODNiNHwwfDB8NjM4NjcxMTMwMDkyNzI4OTE3fFVua25vd258VFdGcGJHWnNiM2Q4ZXlKRmJYQjBlVTFoY0draU9uUnlkV1VzSWxZaU9pSXdMakF1TURBd01DSXNJbEFpT2lKWGFXNHpNaUlzSWtGT0lqb2lUV0ZwYkNJc0lsZFVJam95ZlE9PXwwfHx8&sdata=algzSmJzVWs4SXFNNS9jdldtZk0rMnJMbWhNOStGZUVpei9TWkJvQWZMTT0%3d
